# Burr hole locations are associated with recurrence in single burr hole drainage surgery for chronic subdural hematoma^☆^

**DOI:** 10.1016/j.wnsx.2023.100204

**Published:** 2023-04-27

**Authors:** Hiroaki Hashimoto, Tomoyuki Maruo, Yuki Kimoto, Masami Nakamura, Takahiro Fujinaga, Yukitaka Ushio

**Affiliations:** a Department of Neurosurgery, Otemae Hospital, Osaka, Osaka, 540–0008, Japan; b Department of Neurological Diagnosis and Restoration, Graduate School of Medicine, Osaka University, Suita, Osaka, 565–0871, Japan

**Keywords:** Burr hole drainage surgery, Burr hole locations, Chronic subdural hematoma, Quantitative measurement, Recurrence requiring reoperation, DICOM

## Abstract

**Background:**

Various factors have been reported as risk factors for chronic subdural hematomas (CSDH) recurrence. However, few studies have quantitatively evaluated the impact of CSDH locations and burr hole positions on recurrence. This study aimed to reveal the relation between CSDH recurrence and the locations of CSDH and burr holes.

**Methods:**

Initial single burr hole surgeries for CSDH with a drainage tube between April 2005 and October 2021 at Otemae Hospital were enrolled. Patients’ medical records, CSDH volume, and CSDH computed tomography values (CTV) were evaluated. The locations of CSDH and burr holes were assessed using Montreal Neurological Institute coordinates.

**Results:**

A total of 223 patients were enrolled, including 34 patients with bilateral CSDH, resulting in 257 surgeries investigated. The rate of CSDH recurrence requiring reoperation (RrR) was 13.5%. The RrR rate was significantly higher in patients aged ≥76 years, those with bilateral CSDH, and those with postoperative hemiplegia. In RrR, the preoperative CSDH volume was significantly larger, and CTV was significantly smaller. The locations of CSDH had no influence on recurrence. However, in RrR, the locations of burr holes were found to be more lateral and more ventral. Multivariate Cox proportional hazards regression analysis showed that bilateral CSDH, more ventral burr hole positions, and postoperative hemiplegia were risk factors for recurrence.

**Conclusions:**

The locations of burr holes are associated with CSDH recurrence. In RrR, CSDH profiles tend to show a larger volume and reduced CTV. Hemiplegia after burr hole surgery serves as a warning sign for RrR.

## Graphical abstract

**Figure:**
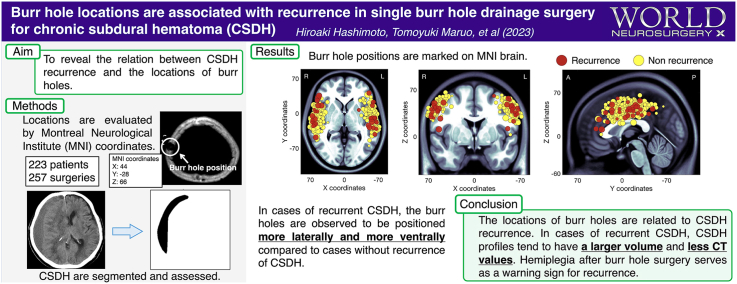

## 1 Introduction

Neurosurgeons frequently encounter chronic subdural hematomas (CSDHs), which occur in approximately 80–120 per 100,000 persons in the aged population^1^, ^2^, ^3^ and are predicted to become the most frequent cranial neurosurgical condition among adults in the future.^1^^,^^4^ Burr hole surgery with a closed drainage system is a simple operation that is effective for CSDH.^5^, ^6^, ^7^, ^8^, ^9^, ^10^, ^11^ However, neurosurgeons sometimes observe CSDH recurrence. Various factors such as age,^12^^,^^13^ and diabetes mellitus^12^ have been reported to be risk factors for recurrence, and recent studies evaluating CSDH quantitatively have revealed that a large volume of preoperative CSDH was a risk factor for recurrence.^14^^,^^15^ Although previous studies involving qualitative assessments of CSDH have reported that hyperdense CSDH^13^^,^^16^^,^^17^ or cranial base CSDH^18^ were associated with a risk of recurrence, the quantitative assessment of CSDH density and its location remains to be performed. Furthermore, the quantitative analysis of burr hole locations is limited. We hypothesized that quantitative assessment of density and locations would enable us to reveal a novel factor for CSDH recurrence.

## 2 Materials and methods

### 2.1 Patients and study setting

In this retrospective study, we enrolled patients who underwent surgical evacuation of CSDHs at Otemae Hospital between April 2005 and October 2021. We only assessed initial surgeries and excluded surgeries for recurrence cases. All cases in this study were diagnosed as CSDH and not hygroma by at least one neurosurgery specialist certified by the Japan Neurosurgical Society. This study was approved by the Ethics Committee of the Otemae Hospital (Osaka, Japan; approval no. CT190122034) and was conducted in accordance with the Declaration of Helsinki for experiments involving humans. Due to the retrospective and noninvasive nature of the study, informed consent was obtained using the opt-out method from our center's website.

### 2.2 Surgical procedure

Surgical evacuation was performed for CSDH, which compressed the brain and induced neurological deficits or medically intractable headaches. Under local anesthesia, a single burr hole was performed, and the dura mater was cut and coagulated. The outer membrane of the CSDH was exposed, and a silicone tube with a closed drainage system was inserted into the subdural space. Irrigation of the hematoma with normal saline and the direction (anterior or posterior) of insertion of the drainage tube were left to the surgeon's discretion. At our institution, a postoperative computed tomography (CT) scan was routinely conducted on the morning following the operation, and the drainage tube was removed within 48 h after surgery. Patients were instructed to remain in the supine position in bed until the tube was removed.

### 2.3 Anticoagulant/antiplatelet medications

Postoperative anticoagulant and antiplatelet medications were temporarily discontinued in patients with a low risk of thrombosis. The medications were resumed once active bleeding was ruled out. The study had no strict criteria for the discontinuation and resumption of anticoagulants or antiplatelets. The decision was left to the surgeon's discretion.

### 2.4 Data collection

We collected scans from CT which was performed preoperatively, and we carefully confirmed that the diagnosis was CSDH, not a subdural hygroma. We defined the density of a subdural hygroma as the same as that of cerebrospinal fluid, and we visually differentiated CSDH from a hygroma. We did not set an exact limit using Hounsfield units for discrimination between CSDH and hygroma. We retrospectively evaluated medical variables related to patients, including sex, age, antiplatelet medication, anticoagulant medication, and pre- and postoperative symptoms such as headache, dementia, aphasia, hemiplegia, gait disturbance, and consciousness level represented by the Glasgow Coma Scale (GCS). Data on hematoma features including hematoma laterality (left or right), bilaterality, presence of midline shift, and subtypes were collected. Number of years since the surgeon had started neurosurgical residency was also recorded. We defined symptoms that were observed until one week after the operation as postoperative symptoms. CSDH were classified into four subtypes: homogeneous, laminar, separated, and trabecular, as described by Nakaguchi et al.^18^ Data related to treatment options, including drainage tube direction (anterior or posterior) and combination with irrigation, were collected. Deterioration based on CT imaging (i.e., increase in hematoma volume) and clinical symptoms (i.e., worsening neurological deficits) necessitated a second operation, defined as recurrence requiring reoperations (RrR). The duration from the first surgery to RrR was recorded.

### 2.5 Quantitatively assessment for CSDH

**Fig. 1:**
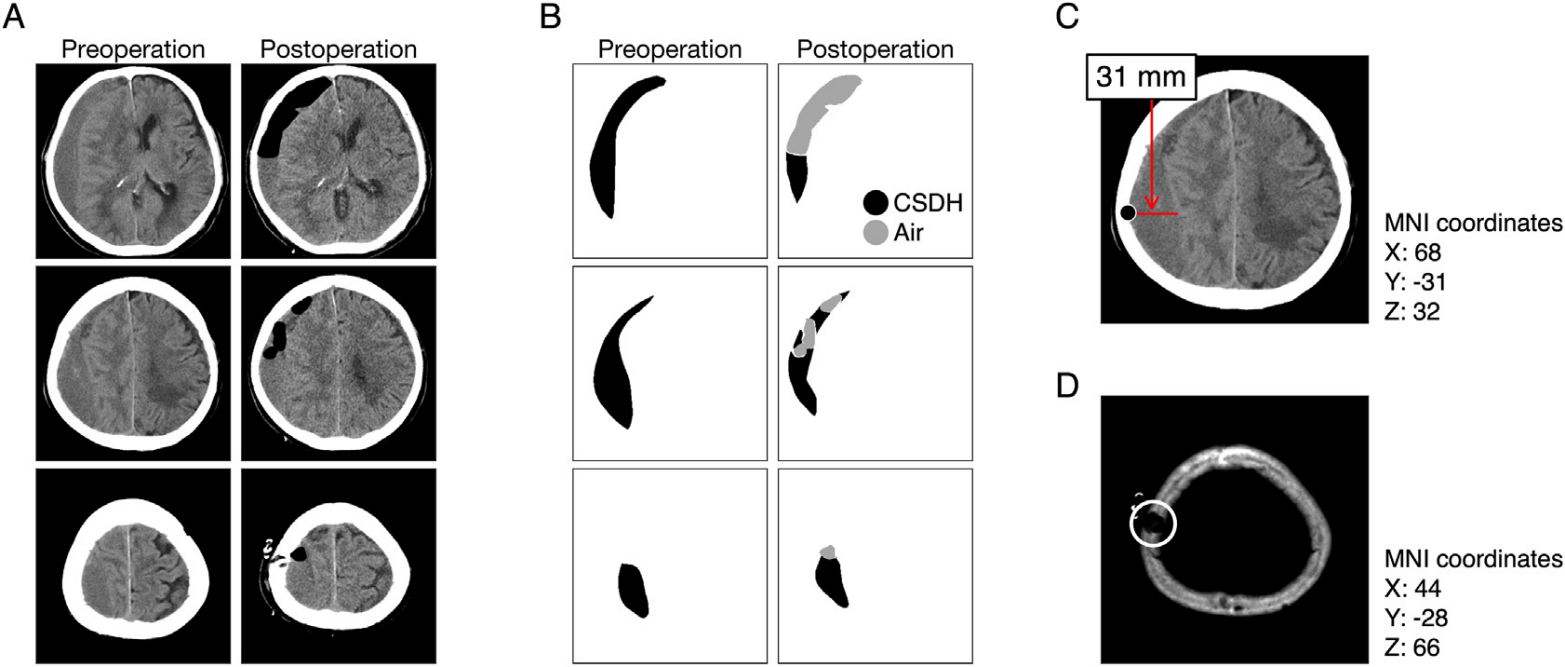
Processing of DICOM CT images of patients with CSDH. (A) CT images of a same CSDH case preoperatively, and postoperatively. (B) Segmentation of CSDH and air are indicated by black and gray colors, respectively. The shown segmentations are acquired from the images in the panel A. (C) The maximum width of CSDH is indicated by a red line, which is 31 mm. We treat this width as the thickness of CSDH. The most lateral position is indicated as a black-filled circle, whose MNI coordinates are shown. This image is the same as the middle preoperative image in the panel A. (D) The position of a single burr hole is indicated as a white circle, whose MNI coordinates are shown. (For interpretation of the references to colour in this figure legend, the reader is referred to the web version of this article.)

Digital imaging and communication in medicine (DICOM) CT images were imported to MATLAB R2020b (MathWorks, Natick, MA, USA), and CSDH and postoperative air were segmented manually using the image segmenter app in MATLAB (https://www.mathworks.com/help/images/ref/imagesegmenter-app.html; Fig. 1A and B). This method is used in our other study.^19^ These procedures enabled us to calculate the volume and average CT values (CTVs) of the segmented regions. We calculated the volume of CSDH preoperatively, and postoperatively, and the volume of postoperative air. The averaged CTVs were calculated from preoperative CSDH segmentations, and the standard deviation (SD) was also calculated. The drainage volume was the difference obtained from the preoperative CSDH volume minus the postoperative CSDH volume. The drainage ratio was calculated by dividing the drainage volume by the preoperative CSDH volume. We defined the thickness (mm) of preoperative CSDH as the maximum horizontal width of preoperative CSDH segmentations (Fig. 1C). The homogeneous subtypes were divided into three types according to the average CTV: hypodensity <30, 30 ≤ isodensity <40, and 40 ≤ hyperdensity.

DICOM CT images were converted into the standard brain using the Montreal Neurological Institute (MNI) coordinates using Brainstorm software (http://neuroimage.usc.edu/brainstorm/). The MNI coordinates for the most lateral position of the CSDH thickness (indicated by a black dot in Fig. 1C) and burr hole (indicated by a white circle in Fig. 1D) were collected. We treated the thickness positions as a representation of the CSDH locations. We changed the X coordinates to absolute values for the evaluation. The relative values of distance between the thickness of the CSDH and the burr hole was calculated using the following equation:

$$Distance=\sqrt[{2}]{{\left(x_{thickness}-x_{burr\;hole}\right)}^{2}+{\left(y_{thickness}-y_{burr\;hole}\right)}^{2}+{\left(z_{thickness}-z_{burr\;hole}\right)}^{2}}$$

### 2.6 Statistical analyses

Categorical data and continuous variables were presented as frequencies (percentages) or mean ± SD, respectively. Clinical differences between non-RrR (nRrR) and RrR were assessed using the chi-squared test for categorical variables. Since the distribution of continuous variables was a non-parametric distribution, we used the two-tailed Wilcoxon rank-sum test for continuous variables. The Kruskal–Wallis test was used to compare the four subtype groups. We calculated the Spearman correlation coefficients between the continuous variables.

The receiver operating characteristic (ROC) curve was used to evaluate predictors of RrR. The cut-off value was defined as the maximal Youden index (sensitivity ​+ ​specificity - 1). Kaplan–Meier survival curves were plotted (https://github.com/dnafinder/kmplot, Curve Cardillo G, 2008), and the log-rank test was conducted to compare the time to recurrence (https://github.com/dnafinder/logrank, Cardillo G. 2008). Bonferroni correction was used for solution of multiple comparisons.

Univariate Cox proportional hazards regression analyses were used to calculate hazard ratios (HRs) with 95% confidence intervals (CIs) for RrR. The variables analyzed in the regression analyses were potential predictors, and all of them showed a *p*-value <0.05, as determined by the chi-squared test or Wilcoxon rank-sum test. Multivariable Cox proportional hazards regression was performed to ensure that the variables were independently predictive of RrR. Statistical analyses were performed using the Statistical and Machine Learning Toolbox of MATLAB R2020b.

### 2.7 Bilateral CSDH

Bilateral CSDH surgeries were counted as two different surgeries. In bilateral CSDH onset, almost patients underwent bilateral burr hole surgeries simultaneously; in one patient with bilateral CSDH onset, only one side was treated (Supplementary Fig. 1A). The factors that were one value per patient, such as age, sex, and symptoms, were statistically handled based on patients. However, the factors that were one value per hematoma, such as CSDH volume and CT values, were statistically handled based on surgeries. In Cox proportional hazards regression analyses, we treated bilateral CSDH cases based on patients. If the right and left hematoma of bilateral CSDH showed simultaneous recurrence or no recurrence, we used averaged values of CSDH volume, CTV, and MNI coordinates for Cox proportional hazards regression analyses. If bilateral CSDH showed hemilateral recurrence, we used values obtained from the recurrence side (Supplementary Fig. 2).

### 2.8 Data availability

All data in this study are available from the corresponding authors upon reasonable request and after additional ethics approval.

## 3 Results

### 3.1 Baseline characteristics

Data on a total of 257 surgeries performed in 223 patients were collected (Supplementary Fig. 1A). In 37 cases, postoperative clinical information was not available, and in 38 cases, preoperative clinical information was not available (Supplementary Fig. 1B). Since there was no information about irrigation in 43 surgeries, irrigation data were collected from 214 surgeries (Supplementary Fig. 1C). Otemae Hospital did not have an electronic medical records system prior to 2007, and paper-based medical records from 2005 until 2007 could not be obtained. All cases underwent single burr hole surgery. No patients have undergone other surgical operations for CSDH, such as twist drill, two burr holes, craniotomy, and middle meningeal artery embolization, in Otemae Hospital.

Of the 223 patients (mean age 76.9 years; range 49–95 years; 59 females, 26.5%), 30 patients (13.5%) experienced RrR, and the median interval between initial surgery and RrR was 31 days. Notably, there were four cases where RrR occurred within one week after surgery: two cases on postoperative day (POD) 1, one case on POD 2, and one case on POD7.

Descriptive results based on patients are shown in Table 1. Over 76 years old, bilateral CSDH, and postoperative hemiplegia were significantly associated with RrR. A prescription of anticoagulant and/or antiplatelet medication and preoperative symptoms did not influence RrR.

**Table 1:** Descriptive statistics regarding demographic characteristics, and pre- and postoperative clinical information calculated from 223 patients. *P* values are calculated using the chi-squared test for categorical variables, and the Wilcoxon rank-sum test for continuous variables. Statistically significant *p* values are flagged with an asterisk.

Characteristics analyzed	Patients n (%)	nRrR (%)	RrR (%)	*p*-value
Sex				
Male	164 (74)	141 (86)	23 (14)	0.68
Female	59 (26)	52 (88)	7 (12)	
**Mean age ± SD (years)**				
Male	75.8 ± 10.4	75.2 ± 10.4	79.0 ± 9.4	0.10
Female	79.9 ± 8.3	79.2 ± 8.5	84.6 ± 5.0	0.10
**Age categories (years)**				
≤76	85 (38)	79 (93)	6 (7)	**∗0.028**
>76	138 (62)	114 (83)	24 (17)	
**Bilateral CSDH**				
Yes	35 (16)	26 (74)	9 (26)	**∗0.021**
No	188 (84)	167 (89)	21 (11)	
**Laterality (188 hemilateral onset)**				
Left	98 (52)	86 (88)	12 (12)	0.63
Right	90 (48**)**	81 (90)	9 (10)	
**Midline shift**				
Yes	173 (78)	152 (88)	21 (12)	0.29
No	50 (22)	41 (82)	9 (18)	
**Career of surgeons (years)**		9.7 ± 4.5	9.9 ± 4.9	0.83
**Clinical information before operation ∗1**				
**Headache**				
Yes	40 (22)	32 (8)	8 (20)	0.46
No	145 (78)	123 (85)	22 (15)	
**Dementia**				
Yes	39 (21)	31 (79)	8 (21)	0.41
No	146 (79)	124 (85)	22 (15)	
**Aphasia**				
Yes	9 (5)	7 (78)	2 (22)	0.62
No	176 (95)	148 (84)	28 (16)	
**Hemiplegia**				
Yes	126 (68)	107 (85)	19 (15)	0.54
No	59 (32)	48 (81)	11 (19)	
**Gait disturbance**				
Yes	159 (86)	133 (84)	26 (16)	0.90
No	26 (14)	22 (85)	4 (15)	
**GCS**				
15	107 (58)	94 (88)	13 (12)	0.079
3–14	78 (42)	61 (78)	17 (22)	
**Anticoagulant**				
Yes	24 (13)	22 (92)	2 (8)	0.26
No	161 (87)	133 (83)	28 (17)	
**Antiplatelet**				
Yes	37 (20)	32 (86)	5 (14)	0.62
No	148 (80)	123 (83)	25 (17)	
**Both anticoagulant and antiplatelet**				
Yes	6 (3)	6 (100)	0 (0)	0.27
No	179 (97)	149 (83)	30 (17)	
**Either anticoagulant or antiplatelet**				
Yes	55 (30)	48 (87)	7 (13)	0.40
No	130 (70)	107 (82)	23 (18)	
**Clinical information after operation ∗2**				
**Complication**				
Yes	12 (6)	9 (75)	3 (25)	0.39
No	174 (94)	147 (84)	27 (16)	
**Dementia**				
Yes	28 (15)	22 (79)	6 (21)	0.41
No	158 (85)	134 (85)	24 (15)	
**Hemiplegia**				
Yes	5 (3)	0 (0)	5 (100)	**∗2.4** × **10**^**−**^**^7^**
No	181 (97)	156 (86)	25 (14)	
**Gait disturbance**				
Yes	19 (10)	13 (68)	6 (32)	0.053
No	167 (90)	143 (86)	24 (14)	
**GCS score**				
15	150 (81)	128 (85)	22 (15)	0.27
3–14	36 (19)	28 (78)	8 (22)	

∗1: 185 patients are analyzed.

∗2: 186 patients are analyzed. These are indicated in Supplementary Fig. 1B.

### 3.2 Quantitative measurement

Descriptive results based on surgeries are shown in Table 2. Preoperative, and postoperative CSDH volume of RrR was significantly larger than that of nRrR (*p* = 0.024, and 0.00036, respectively; Wilcoxon rank-sum test). Contrarily, the CTV of preoperative CSDH and the drainage ratio were significantly lower in RrR than in nRrR cases (*p* = 0.010, and 0.027, respectively; Wilcoxon rank-sum test). Preoperative CSDH thickness, SD values of CTV, and postoperative air volume showed no significant differences.

**Table 2:** Descriptive statistics regarding operative information, and quantitative measures using DICOM CT images calculated from 257 surgeries. P values are calculated by the chi-squared test for categorical variables, the Wilcoxon rank-sum test for continuous variables, and the Kruskal–Wallis test for the four subtype groups. Statistically significant p values are flagged with an asterisk.

Characteristics analyzed	Patients n (%)	nRrR (%)	RrR (%)	*p*-value
**Preoperative CSDH volume ± SD (ml)**		116.1 ± 36.6	134.0 ± 38.8	**∗0.024**
**Preoperative CSDH thickness ± SD (mm)**		28.0 ± 5.1	28.6 ± 6.9	0.90
**Preoperative CTV ± SD**		34.4 ± 6.9	31.1 ± 7.7	**∗0.010**
**Preoperative SD of CTV ± SD**		9.6 ± 2.6	10.3 ± 2.7	0.12
**Subtype**				
Homogeneous	104 (40)	89 (86)	15 (14)	0.053
Laminar	16 (6)	13 (81)	3 (19)	
Separate	64 (25)	52 (81)	12 (19)	
Trabecular	73 (28)	70 (96)	3 (4)	
**Subtype CTV ± SD**				
Homogeneous	34.8 ± 8.9			**∗0.024**
Laminar	35.6 ± 5.9			
Separate	33.7 ± 5.6			
Trabecular	32.8 ± 5.1			
**SD of subtype CTV ± SD**				
Homogeneous	8.2 ± 1.9			**∗1.3** × **10**^**−**^**^17^**
Laminar	9.6 ± 2.6			
Separate	11.9 ± 2.4			
Trabecular	9.9 ± 2.2			
**Sub-grading in homogeneous group ∗1**				
Hypodensity (CTV <30)	25 (24)	18 (72)	7 (28)	0.082
Isodensity (30 ≦ CTV <40)	45 (43)	40 (89)	5 (11)	
Hyperdensity (CTV ≧ 40)	34 (33)	31 (91)	3 (9)	
**MNI coordinates of thickness ± SD**				
X (absolute values)		53.4 ± 9.9	51.8 ± 11.5	0.52
Y		1.8 ± 39.8	12.2 ± 39.4	0.16
Z		29.5 ± 18.2	28.5 ± 16.5	0.60
**MNI coordinate of burr holes ± SD**				
X (absolute values)		55.3 ± 7.3	58.2 ± 7.3	**∗0.012**
Y		−5.4 ± 18.0	−5.6 ± 22.2	0.62
Z		43.6 ± 8.9	39.2 ± 11.1	**∗0.019**
**Distance ± SD**		41.4 ± 24.3	43.3 ± 27.5	0.93
**Postoperative CSDH volume ± SD (ml)**		47.2 ± 28.2	68.2 ± 34.1	**∗0.00036**
**Drainage ratio between pre- and postoperative ± SD**		0.59 ± 0.22	0.48 ± 0.23	**∗0.027**
**Postoperative air ± SD (ml)**		11.2 ± 18.6	11.0 ± 11.7	0.17
**Drainage tube direction**				
Anterior	110 (43)	93 (85)	17 (15)	0.28
Posterior	147 (57)	131 (89)	16 (11)	
**Irrigation ∗2**				
Do	60 (28)	52 (87)	8 (13)	0.60
Undo	154 (72)	129 (84)	25 (16)	

∗1: 104 surgeries classified into the homogenous subtype are analyzed.

∗2: 214 surgeries are analyzed, which are indicated in Supplementary Fig. 1C.

**Fig. 2:**
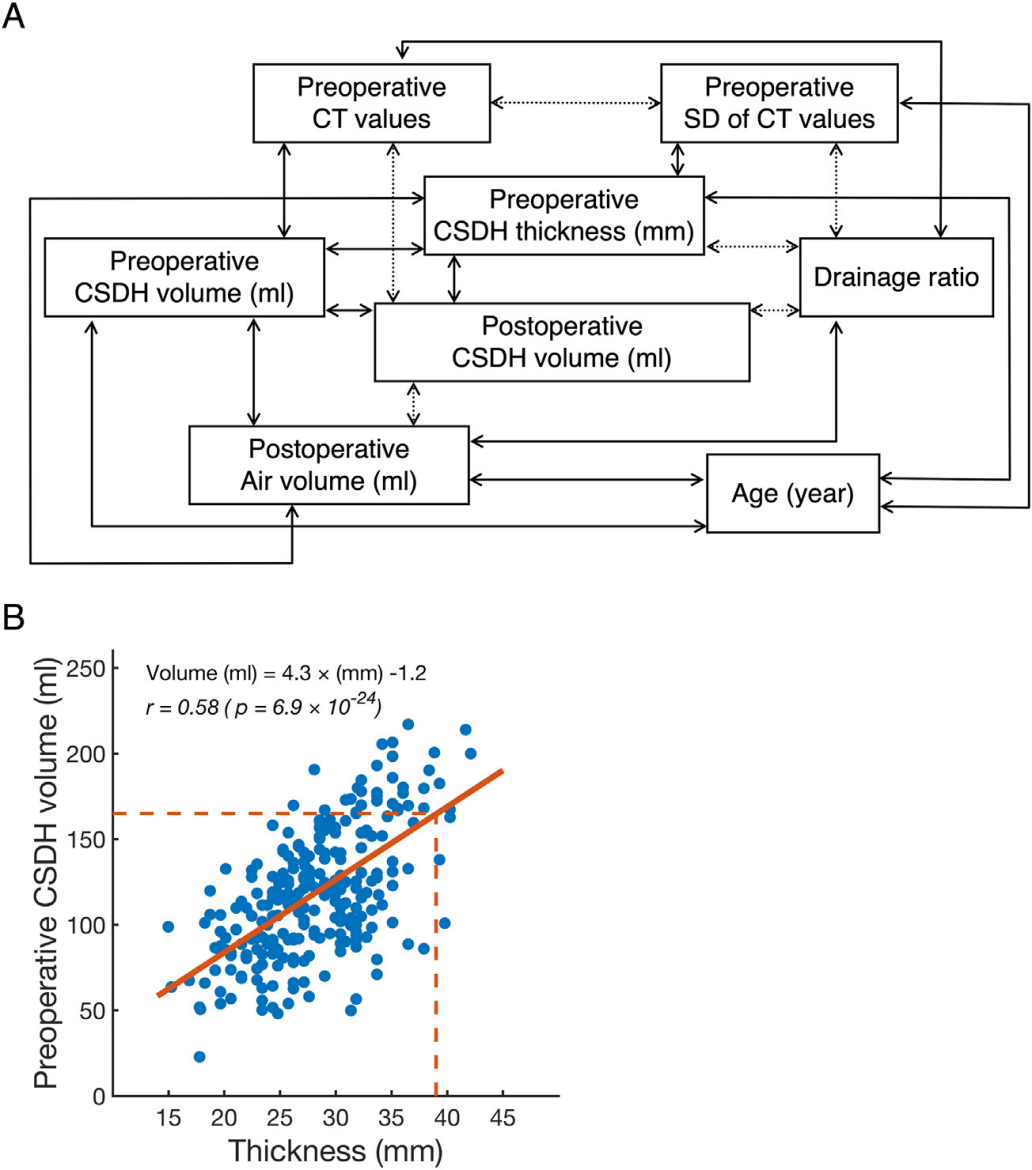
Correlation relations between quantitative variables. (A) Correlation relations that indicate *p* values < 0.05 are shown, calculated based on 257 surgeries. Positive and negative correlations are indicated as a solid and a broken line. (B) Scatter plot between preoperative CSDH volume and thickness are shown. The regression line is indicated as a red line and *r* is the correlation coefficient. Red dashed lines correspond to 39 mm and 165 ml. (For interpretation of the references to colour in this figure legend, the reader is referred to the web version of this article.)

In Fig. 2A, preoperative CSDH volume was positively correlated with postoperative CSDH volume, CSDH thickness, age, air volume, and CTV. The drainage ratio and air volume showed a negative correlation with the postoperative CSDH volume. There is also a negative correlation between CTV and SD of CTV, indicating that higher hematoma density resulted in more homogeneous density. Furthermore, postoperative CSDH volume showed a negative correlation with CTV, suggesting that thicker CSDH concentration led to smaller postoperative CSDH volume. The regression line between preoperative CSDH volume and thickness was defined by the following equation: preoperative CSDH volume (ml) = 4.3 × thickness (mm) −1.2 (Fig. 2B).

In the four CSDH subtypes, there was no statistical difference in RrR, but the laminar and separate types showed highest recurrence rates (19% and 19%, respectively) and the trabecular type showed lowest recurrence rates (4%). There were significant differences in CTV and SD of CTV between the four subtypes (*p* = 0.024, and 1.3 × 10^−17^, respectively; Kruskal–Wallis test). In the homogeneous subtype, the hypodense hematoma was associated with a higher recurrence rate (28%) than the isodense (11%) and hyperdense hematomas (9%), but the difference was not significant.

### 3.3 Locations of CSDH and burr hole

**Fig. 3:**
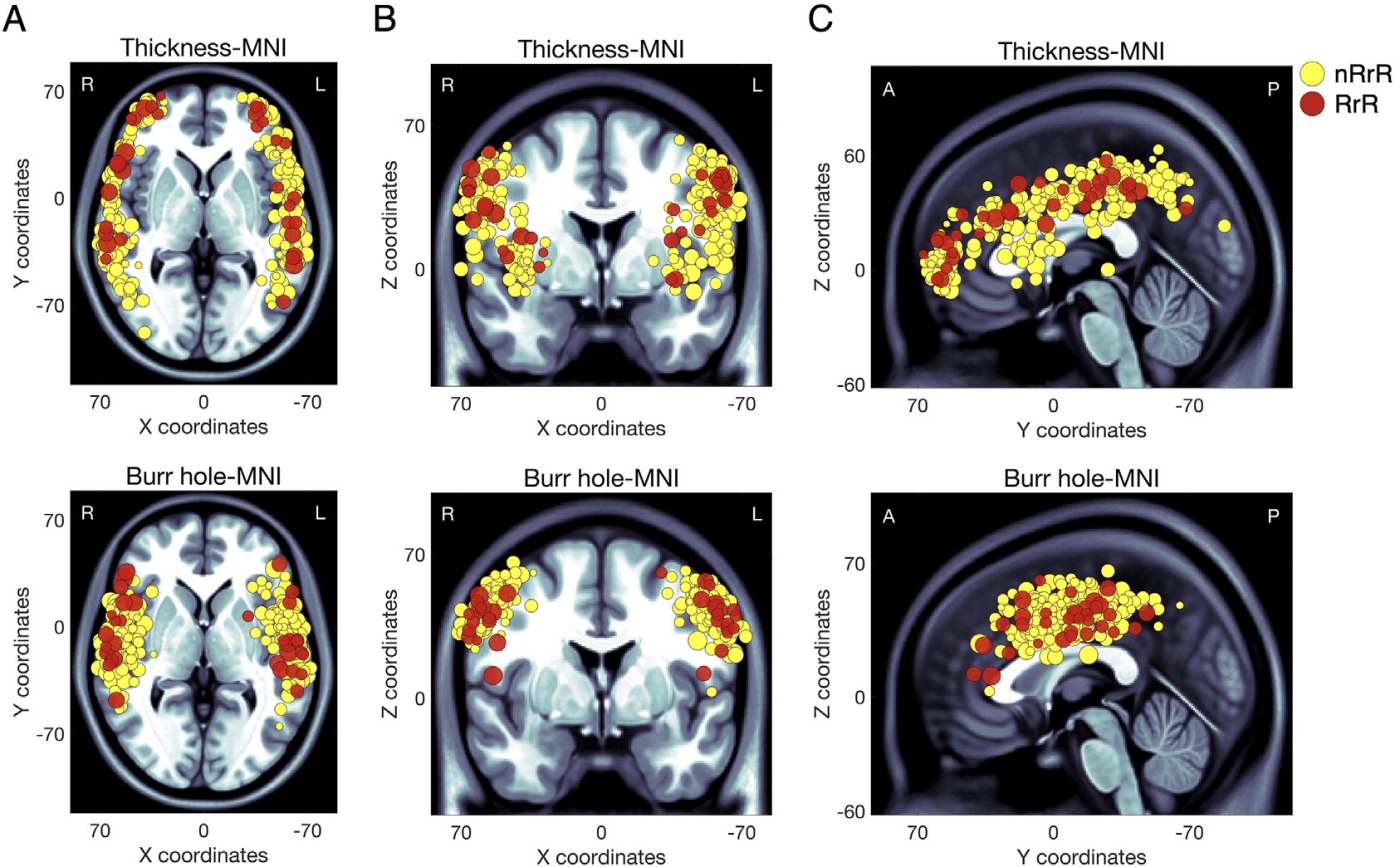
Distribution of CSDH thickness and burr hole positions. According to MNI coordinates, the thickness position of CSDH (upper row) and burr hole position (lower row) are plotted over the MNI standard brain. Cases of recurrence RrR and nRrR are indicated as red and yellow circles, respectively. The circles are scaled linearly to the preoperative CSDH volume. Axial slices (X–Y plane), coronal slices (X-Z plane), and sagittal slices (Y-Z plane) are indicated in panels A, B, and C. R, right; L, left; A, anterior; P, posterior.

**Fig. 4:**
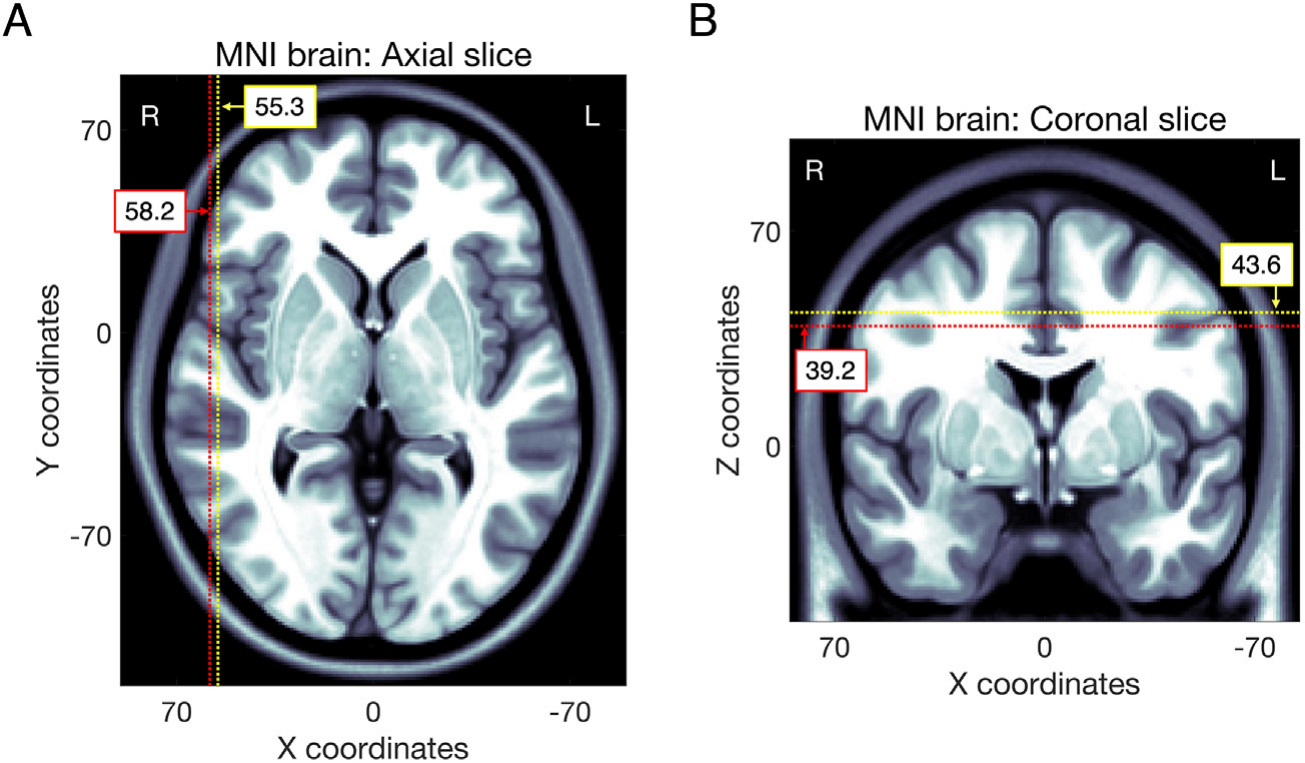
Burr holes-related X and Z coordinates indicating significant differences between RrR and nRrR. The X and Z burr hole positions indicating significant differences in Table 2 are shown on the MNI standard brain in the A and B panels, respectively. The positions related to RrR are indicated by red dashed lines. The positions related to nRrR are indicated by yellow dashed lines.

In the MNI coordinates of the thickness, there were no statistical differences between RrR and nRrR cases (Table 2). All thickness and burr hole positions were plotted over the standard brain (Fig. 3). The distribution patterns of thickness positions varied widely in both nRrR and RrR cases. In contrast, the X and Z MNI coordinates of burr hole positions showed significant differences. The X was higher (more lateral), and the Z was lower (more ventral) in RrR (*p* = 0.012, and 0.019, respectively; Wilcoxon rank-sum test) (Figs. 3 and 4).

### 3.4 Follow-up outcomes

**Fig. 5:**
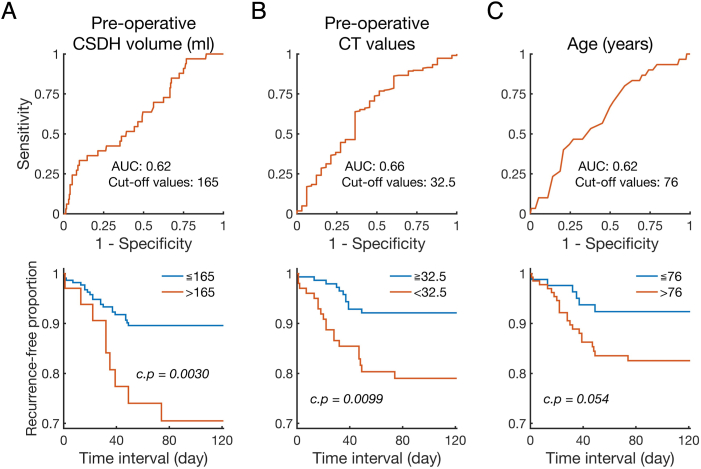
ROC and Kaplan–Meier curves. In the upper row, ROC curves are indicated. In the lower row, Kaplan–Meier curves are indicated. Quantitative variables include preoperative CSDH volume (A), CT values (B), and age (C). Area under the curve (AUC) and cut-off values are shown. Kaplan–Meier curves have been plotted using these cut-off values. The results of CSDH volume and CT values are calculated based on 257 surgeries, and the results of age were calculated based on 223 patients. *P* values from the log-rank test are indicated and corrected by Bonferroni correction. c.p, corrected *p* values.

According to the Youden index, the ROC analysis showed that the optimal cut-off values were 165 ml for preoperative CSDH volume, 32.5 for CTV, and 76 years for age (Fig. 5, upper row). The Kaplan–Meier curve using these cut-off values showed that most RrR cases occurred within approximately 80 days. Log-rank tests revealed that larger preoperative CSDH volume, and lesser CTV were associated with a significant risk of RrR (corrected *p* = 0.0030, and corrected *p* = 0.0099 with Bonferroni correction, respectively; Fig. 5, lower row).

### 3.5 Risk factors for recurrence of CSDH

Preoperative CSDH volume, age, bilateral CSDH, and postoperative hemiplegia were positively associated with RrR, as revealed by univariate Cox proportional hazards regression analyses. Preoperative CTV and Z coordinates of burr hole positions were inversely associated with RrR. Multivariate Cox proportional hazards regression analyses showed that bilateral CSDH (HR 2.55, *p* = 0.035), Z coordinates of burr hole positions (HR 0.96, *p* = 0.025), and postoperative hemiplegia (HR 17.43, *p* = 0.000012) were independent risk factors for RrR (Table 3).

**Table 3:** Univariate and multivariate Cox proportional hazards regression analyses of characteristics relate to postoperative recurrence requiring reoperation for CSDH. The 186 patients indicated in Supplementary Fig. 1B are used. Statistically significant p values are flagged with an asterisk.

	Cox proportional hazards regression analyses			
Characteristics analyzed	Univariate analysis		Multivariate analysis	
	HR (95% CI)	*p-*value	HR (95% CI)	*p-*value
Preoperative CSDH volume (ml)	1.01 (1.00–1.02)	**∗0.032**	1.00 (0.99–1.01)	0.59
Preoperative CT values	0.94 (0.90–1.00)	**∗0.035**	0.98 (0.92–1.04)	0.45
Age (years)	1.05 (1.01–1.10)	**∗0.024**	1.02 (0.98–1.07)	0.33
Bilateral CSDH	2.45 (1.11–5.37)	**∗0.026**	2.55 (1.07–6.07)	**∗0.035**
X coordinate of burr holes	1.06 (1.00–1.12)	0.051	–	–
Z coordinate of burr holes	0.95 (0.92–0.99)	**∗0.0087**	0.96 (0.92–0.99)	**∗0.025**
Postoperative hemiplegia	17.66 (6.52–47.82)	**∗0.000000016**	17.43 (4.84–62.76)	**∗0.000012**

## 4 Discussion

We hypothesized that quantitative assessment of density and locations would enable us to reveal a novel factor for CSDH recurrence. By assessing quantitative values acquired from DICOM CT images of CSDH patients who underwent an initial single burr hole surgery with a closed drainage system, we could show that CTV was significantly lower in RrR cases than in nRrR cases, and a hypodense subgroup (CTV <30) of homogeneous CSDH cases were associated with a higher RrR rate than iso- and hyperdense subgroups. Regression analyses showed that higher CTVs in CSDHs were associated with a lower risk of RrR. This result is novel, contrary to the results of previous studies indicating that hyperdense hematoma was associated with recurrence.^13^^,^^14^^,^^16^^,^^17^

We inferred that our results might be related to the poor surgical outcomes associated with hygroma. A subdural hygroma is thought to be a collection of cerebrospinal fluid without blood due to traumatic arachnoid tearing,^18^^,^^20^ and CT images of hygroma show hypodensity.^21^^,^^22^ Nearly 50% of patients with a subdural hygroma develop CSDH,^20^ and the effect of surgery for hygroma is poor.^23^^,^^24^ Although previous studies assessed CSDH density qualitatively, our study assessed CSDH quantitatively. Furthermore, we successfully demonstrated statistical differences among the four subtype of CSDH using CTV and its SD. Additionally, we found that the laminar and separate types showed the highest recurrence rates, while the trabecular type showed the lowest recurrence rates, which is consistent with the findings of a previous study.^18^ These results support the correctness and reliability of our quantitative analyses.

We demonstrated that more lateral and more ventral burr hole positions were associated with RrR. According to our result, we propose that making a burr hole located a bit more parietal to the temporal line, avoiding the temporal muscle, would be effective for reducing RrR. Empirically, burr holes were created overlying the hematoma and were mainly on the temporal line, ranging in location from the frontal to the temporal lobe.^6^ However, previous studies assessing burr hole positions quantitatively and the relation between burr hole positions and RrR have been lacking. Due to the wide variability in head size among individuals, the positions could not be compared and analyzed based on absolute values such as millimeters. Therefore, we relied on relative values, such as MNI coordinates, to assess burr holes positions and CSDH locations.

In the bottom panel of Fig. 3A, it appears that burr holes in RrR exhibit two clusters: one in the anterior/pterion group and another in the posterior/temporal group. We infer that a middle meningeal artery (MMA) passes through the area between these two clusters. MMA embolization has been shown to be a safe and effective alternative to conventional surgical techniques,^25^ and we speculate that in cases where the MMA was coagulated concurrently with dura matter coagulation, the risk of RrR may be reduced. Further analyses are needed to elucidate the association between burr holes positions and RrR.

In this study, 13.5% of the participants had RrR, and most CSDHs recurred within 80 days. These findings were concordant with previous studies.^12^^,^^13^^,^^17^^,^^26^ Several factors have been reported to influence recurrence of CSDH, including age, sex, use of antithrombotic agents such as anticoagulants and antiplatelets, diabetes mellitus, smoking, bilateral CSDH, midline shift, GCS score at admission, and high-density lipoprotein levels.^12^^,^^13^^,^^16^^,^^26^, ^27^, ^28^, ^29^ We also demonstrated that age and bilateral CSDH influenced recurrence, in agreement with the results of previous studies. Since the career years of operating surgeons was not significantly different between nRrR and RrR cases, we infer that a burr hole surgery for CSDH is a safe procedure for neurosurgical residents to perform. The recurrence rate of cranial base CSDH was reported to be high.^18^ However, our results demonstrated that the location of CSDH did not influence recurrence. Additionally, we demonstrated that if patients showed postoperative hemiplegia even after burr-hole surgery, this symptom was highly associated with RrR. If hemiplegia continues after the operation, it is reasonable that neurosurgeons try to perform re-operation for relief of the symptom.

Preoperative CSDH volume was a factor for recurrence.^13^, ^14^, ^15^ We showed that a large volume of preoperative CSDH was associated with recurrence and the optimal cut-off value for preoperative CSDH volume was 165 ml. However, in a clinical setting, it is difficult to determine CSDH volume easily and rapidly using DICOM CT images, in contrast to the thickness of the CSDH. Therefore, we showed the regression line and calculated the thickness (39 mm) corresponding to 165 mL (Fig. 2B). Therefore, we propose that physicians monitor for possible recurrence in cases of CSDH with thicknesses of over 39 mm.

Preoperative CSDH volume was positively correlated with several parameters (Fig. 2A). Postoperative CSDH volume,^14^^,^^30^ CSDH thickness,^7^^,^^16^^,^^31^ and postoperative air volume^32^^,^^33^ were associated with recurrence. However, all three of these parameters were positively correlated with preoperative CSDH volume. Since preoperative CSDH volume showed a positive correlation with postoperative air volume, we infer that larger CSDH volumes tend to have larger air volumes after burr hole surgery. Therefore, we believe that the true factor influencing recurrence is the preoperative CSDH volume.

Although postoperative air volume was previously thought to be a risk factor for recurrence,^32^^,^^33^ our results showed that postoperative air volume was not a risk factor for recurrence and positively correlated with preoperative CSDH volume. It is reasonable to postulate that the larger the preoperative CSDH volume, the higher the recurrence rates, and the larger the postoperative air volume. Persistence of subdural air after surgery has been significantly correlated with poor brain re-expansion,^34^ and patients with residual subdural air seven days after surgery have been shown to have a higher recurrence rate than those without air.^35^ Moreover, poor brain re-expansion seven days after surgery has been shown to be a significant risk factor for recurrence,^26^ and cerebral atrophy is also associated with the development of CSDH.^36^ Sufficient subdural space is thought to be a prerequisite for developing CSDH.^20^ Therefore, we infer that a true risk factor for recurrence of CSDH may be poor re-expansion of the brain or large preoperative CSDH volume, and postoperative air volume may be the result, and not a cause, of poor brain re-expansion.

As for the surgical technique, twist drill craniostomy and double burr hole craniostomy may be performed in the management of CSDH.^6^ However, we think that these operations are considered minor in Japan, and the single burr hole is the surgery of choice for almost all cases of CSDH. In our institution, all cases of CSDH were treated by single burr hole surgery. Furthermore, the technical aspects of the single burr hole surgeries did not vary during the study period, as confirmed by our operative notes. Some previous studies have proposed that a drainage tube should be inserted into the frontal convexity to remove subdural air.^35^ However, our results showed that the direction of the drainage tube had no influence on recurrence. This result also supports the notion that postoperative air is not a risk factor for recurrence. There have been conflicting reports showing irrigation may correlate to recurrence.^9^ However, our results showed no statistically significant effectiveness of irrigation.

This study has some limitations. We focused on only single burr hole surgery, not other types of surgery, such as two-burr hole surgeries or twist drill surgery.^6^ Thus, the results of this study can be applied to only single burr hole surgeries. Second, there may also be a possibility that the surgical procedure was not consistently performed because we collected long-term data spanning 17 years (2005–2021). However, we believe that neurosurgeons in Japan normally treat CSDH through single burr hole and drainage surgery, as opposed to other types of surgery, and the surgical techniques involved in the procedure had already been established in 2005. Therefore, we were not concerned about the biases which may arise due to changes in the surgical procedure over 15 years. Third, due to the retrospective nature of the study, there were no concrete criteria for the use of anticoagulant or antiplatelet medications. Additionally, there were no specific criteria regarding the direction (anterior or posterior) of a silicon tube insertion or whether irrigation of the hematoma should be performed or not. This lack of standardized protocols hinders the precise assessment of the influence of these medications and surgical procedures on recurrence. To solve these problems, we believe that a prospective study would be needed. Fourth, we routinely conducted follow-up on almost all patients who underwent surgical evacuation of CSDHs for a few months. However, some outpatients discontinued their follow-up due to private or social issues. As a result, there is a possibility that we may have missed a recurrence. Fifth, we only focused on patients with CSDH who underwent surgical operations. However, some outpatients with CSDH were treated with medications such as Gorei-san, Chinese herbal medicine, and avoided surgery. We prescribed Gorei-san to some patients who underwent surgery for CSDH, and there is a possibility that the use of Gorei-san may have prevented recurrence. However, it should be noted that the effectiveness of Gorei-san for preventing recurrence of CSDH has not been proven.^37^^,^^38^ Additionally, Tranexamic acid is being investigated as a medication for CSDH,^39^ but we did not include analysis of such medications in this study. Finally, since burr holes were made in the area that neurosurgeons thought that they were effective for drainage, there would be some bias in the results of MNI location of burr holes.

## 5 Conclusion

We hypothesized that quantitative assessment of density and locations would enable us to reveal a novel factor for CSDH recurrence. Preoperative CSDH volume, CTV of the hematoma, and more ventral burr hole positions were related to CSDH recurrence. In cases of low concentration and large volume CSDH, attention should be paid to recurrence.

## Credit author statement

**Hiroaki Hashimoto:** Conceptualizaion, Methodology, Software, Formal analysis, Investigation, Data Curation, Writing - Original Draft, Writing - Review & Editing, Visualization, Project administration, Funding acquisition. **Tomoyuki Maruo:** Investigation, Resources, Writing - Review & Editing, Supervision, Project administration. **Yuki Kimoto:** Investigation. **Masami Nakamura:** Investigation. **Takahiro Fujinaga:** Investigation. **Yukitaka Ushio:** Supervision.

## Contributorship statement

All authors included on this paper fulfill all criteria of authorship including substantial contributions to the conception or design of the work, acquisition, analysis, and interpretation of data, drafting or revising the work for intellectually important content, final approval of the version to be published, and agreement to be accountable for all aspects of the work, including ensuring that questions regarding the accuracy or integrity of the work are appropriately addressed and resolved.

## Sources of funding

The 10.13039/501100001691Japan Society for the Promotion of Science (JSPS) KAKENHI [JP21K16629 (Hiroaki Hashimoto)] supported this work.

## Declaration of competing interest

The authors report no conflict of interest.
