# Supplementary material for: Burr hole locations are associated with recurrence in single burr hole drainage surgery for chronic subdural hematoma
Source: World Neurosurg X. 2023 Apr 27;19:100204. doi: 10.1016/j.wnsx.2023.100204 (PMC10189492; doi:10.1016/j.wnsx.2023.100204)
Supplement: Multimedia component 1 [file mmc1.pdf]

Burr hole locations are associated with recurrence in single burr hole drainage surgery for chronic subdural hematoma.

Supplementary Figure 1
Supplementary Figure 2

Supplementary Fig.1. Patients and surgeries investigated in this study.

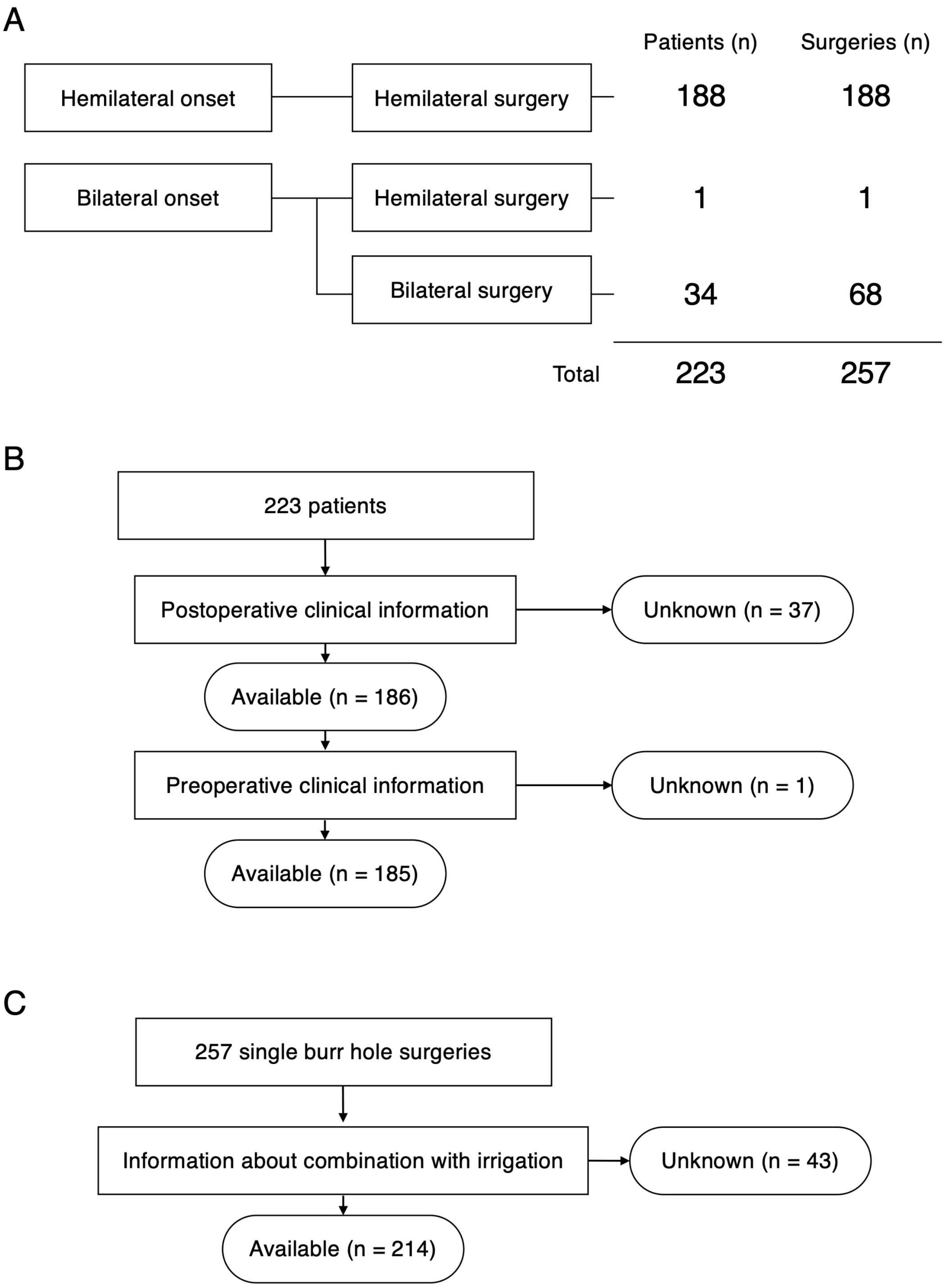

(A) We enroll 223 patients who are suffered from initial onset CSDH including hemilateral onset or bilateral onset. In bilateral onset cases, almost all patients underwent bilateral burr hole surgeries simultaneously. However, one patient undergoes a single side burr hole surgery because the CSDH on the other side does not need to be treated. Since simultaneous bilateral burr hole surgeries are treated as two different surgeries, in total 257 surgeries are enrolled. (B) Flow chart showing 223 patients investigated in this study. (C) Flow chart showing 257 surgeries investigated in this study.

Supplementary Fig.2. How bilateral surgery is handled in Cox proportional hazards regression analyses.

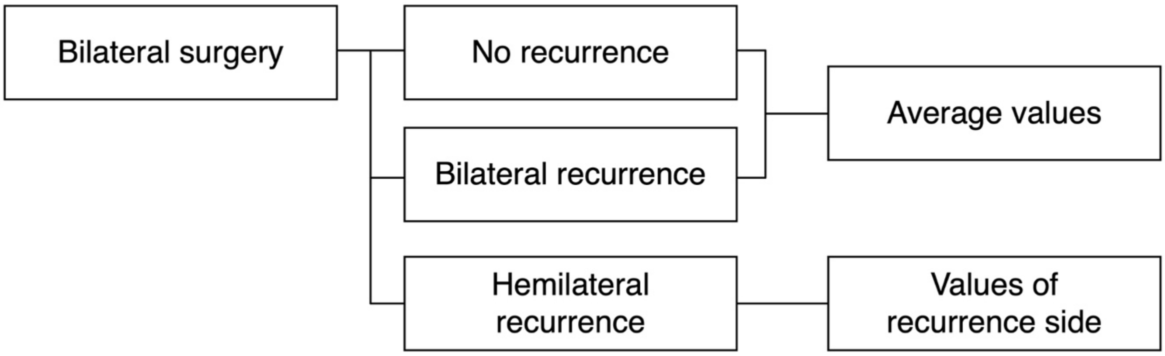

In patients who are undergone simultaneous bilateral surgeries, there are three different outcomes: no recurrence, simultaneous bilateral recurrence, and hemilateral recurrence. In the no recurrence and bilateral recurrence groups, we calculate average values in preoperative CSDH volume, preoperative CT values, and X and Z coordinates of burr holes and use those for Cox proportional hazards regression analyses. In the hemilateral recurrence group, we use values acquired from the recurrence side for Cox proportional hazards regression analyses.
